# Light-Controlled Fruit Pigmentation and Flavor Volatiles in Tomato and Bell Pepper

**DOI:** 10.3390/antiox9010014

**Published:** 2019-12-23

**Authors:** Hee Ju Yoo, Jin-Hyun Kim, Kyoung-Sub Park, Jung Eek Son, Je Min Lee

**Affiliations:** 1 Department of Horticultural Science, Kyungpook National University, Daegu 41566, Korea or; 2 Protected Horticulture Research Institute, National Institute of Horticultural & Herbal Science, RDA, Haman 52054, Korea; weatherboy@korea.kr (J.-H.K.); unicos75@mokpo.ac.kr (K.-S.P.); 3 Department of Horticultural Science, Mokpo National University, Muan 58554, Korea; 4 Department of Plant Science, Seoul National University Seoul, Seoul 08826, Korea; sjeenv@snu.ac.kr

**Keywords:** tomato, pepper, fruit ripening, light, carotenoid, flavonoid, flavor volatile

## Abstract

Light is a major environmental factor affecting the regulation of secondary metabolites, such as pigments and flavor. The Solanaceae plant family has diverse patterns of fruit metabolisms that serve as suitable models to understand the molecular basis of its regulation across species. To investigate light-dependent regulation for fruit pigmentation and volatile flavors, major fruit pigments, their biosynthetic gene expression, and volatiles were analyzed in covered fruits of tomato and bell pepper. Immature covered fruits were found to be ivory in color and no chlorophyll was detected in both plants. The total carotenoid content was found to be reduced in ripe tomato and bell pepper under cover. Naringenin chalcone decreased more than 7-fold in ripe tomato and total flavonoids decreased about 10-fold in immature and ripe pepper fruit under light deficiency. Light positively impacts fruit pigmentation in tomato and bell pepper by regulating gene expression in carotenoid and flavonoid biosynthesis, especially *phytoene synthase* and *chalcone synthase*, respectively. Nineteen volatile flavors were detected, and seven of these exhibited light-dependent regulations for both ripe tomato and pepper. This study will help in improving fruit quality and aid future research works to understand the molecular mechanisms regulating the influence of light-dependency on pigments and flavor volatiles.

## 1. Introduction

Tomato (*Solanum lycopersicum*) and pepper (*Capsicum annuum*) are representative commercial crops highly abundant in nutrients, including carotenoids, flavonoids, and vitamins [1,2]. In particular, carotenoids and flavonoids have important roles in organ pigmentation, accessory photosynthetic pigments, scavenging reactive oxygen species, antioxidant activity, and attracting seed dispersers and pollinators [3,4]. 

Tomato and pepper exhibit similar pigmentation patterns during ripening (i.e., changing from green to red), but their metabolite composition differs significantly. The primary carotenoid in tomato is the acyclic carotenoid *trans*-lycopene, while xanthophyll capsanthin is dominant in pepper [3]. Changes in the expression of carotenoid biosynthetic genes contribute to the accumulation of these carotenoids in ripe tomato and pepper fruit [5]. During ripening, the expression of *phytoene synthase* (*PSY*) and genes encoding desaturase/isomerase are upregulated, while the expression of genes encoding lycopene cyclase is downregulated in tomato [6], leading to the accumulation of lycopene in ripe fruit. *Capsanthin-capsorubin synthase* (*CCS*) is responsible for the synthesis of capsanthin and capsorubin, unique carotenoids in red pepper, from antheraxanthin and violaxanthin, respectively [7]. Tomato and pepper fruit also differ in terms of flavonoid synthesis. Naringenin chalcone is the dominant flavonoid in the ripe fruit of tomato and is mainly synthesized in the peel [8], while pepper fruit accumulates various flavonoids, including quercetin, luteolin, apigenin, and kaempferol; their composition varies by genotype [2]. 

Light is an important environmental factor for organ pigmentation in plants [9]. Light increases the accumulation of carotenoids and flavonoids and accelerates the degradation of chlorophyll in immature fruits [10,11]. Though light is not a primary factor in instigating the synthesis of carotenoids, carotenoid levels under light are higher than those in the dark for *Beta*, *apricot*, and *tangerine* mutants and for red tomatoes [12]. Signaling interactions between light and hormones also significantly affect carotenoid biosynthesis and accumulation [13]. Transcript levels of *ethylene response factor.e4* (*ERF.E4*), a repressor of carotenoid biosynthesis, are lower under light than in the dark. In addition, the transcript levels of *auxin response factor 2a* (*ARF2a*) and *ARF2b*, ripening regulators in tomato fruit, are higher under light [13]. The transcript factor involved in light signal transduction, *phytochrome-interacting factor1* (*PIF1*), represses the expression of *PSY*, leading to reduced carotenoid biosynthesis in tomato fruit [14] under shaded conditions. 

In some crops, light has an important influence on the accumulation of flavonoids in fruit such as grapes and apples [15,16]. For example, anthocyanins accumulate under light but are suppressed in the dark in grapes [15], while the expression of *flavonol synthase4* (*FLS4*), one of the regulatory gene for flavonoids, and *MYB*-transcription factor (*MYBF1*) is significantly lower in grapes under dark conditions, regardless of the temperature [15]. Anthocyanin levels also gradually reduce in the flesh and peel of apples, and flavanol levels are lower in the flesh during fruit ripening in shaded conditions [16]. It has also been found that flavonoid levels, especially those of cyanidin 3-galactoside and quercetin 3-glycoside, are higher in apples located at the top of the tree and in the outer canopy than those found in the inner canopy [17] because the top of the tree and outer canopy generally receive more radiation. The change in the expression of flavonoid biosynthetic genes and transcription factors have also been investigated in bagged and bag-removed red pears. The transcript levels of *chalcone synthase* (*CHS*) and *basic helix-loop-helix* (*bHLH*) were higher in the bag-removed fruit and these levels were correlated with anthocyanin levels [18]. 

Improving the flavor of commercially grown fruit is challenging due to the complexity of evaluation underlying the phenotype. In particular, the genetic regulation of flavor volatile synthesis is not completely understood. Commercial tomato cultivars are typically rated lower in terms of taste and flavor compared to heirloom varieties [19]. The major precursors of flavor volatiles are carotenoids, lipids, and amino acids. In tomato fruit, more than 400 volatiles have been identified; however, only around 20 compounds are believed to be related to the flavor of the fruit [20]. In some cases, environmental factors are more important than genetic factors in terms of biochemical variation. For example, carrots grown at low temperatures tend to be sweeter, while terpenoids and their volatiles, which is responsible for bitter taste, are increased with higher growth temperatures [21]. In pepper, volatile compounds are more strongly affected by environmental variation than by genetic influences [22]. 

Light conditions (e.g., intensity, photoperiod, direction, and quality) are believed to regulate the synthesis of secondary metabolites, including carotenoids and flavonoids [10,11]. However, little is known about the effect of light on the regulation of pigments and flavors in tomato and pepper fruit. In order to understand the effect of light on accumulation of fruit pigments and flavor volatiles in tomato and bell pepper, artificial bagging was done for individual fruit during the period from flowering until the harvesting. Carotenoids and flavonoids coupled with their biosynthetic expressions were analyzed, and volatile compounds were studied. This study is helpful in improving fruit quality and understanding the molecular mechanisms regulating the influence of light-dependency on pigments and flavor volatiles.

## 2. Materials and Methods 

### 2.1. Plant Materials

Tomato (*Solanum lycopersicum* cv. Dafnis,) and bell pepper (*Capsicum annuum* cv. Sirocco,) were grown in a greenhouse in the National Institute of Horticultural and Herbal Science of Rural Development Administration (Haman, Korea). Plants were grown in rock wool media connected to an automatic irrigation system. The supplied nutrient solution was gradually increased from 2.0 to 2.5 dS m^−1^ in terms of electrical conductivity with a pH of 5.8–6.2 according to the plant stage. The nutrient solution including NO_3_ (98 mg L^−1^), NH_4_ (94 mg L^−1^), P (20.6 mg L^−1^), K (156 mg L^−1^), Ca (60 mg L^−1^), and Mg (24.3 mg L^−1^), Fe (2.92 mg L^−1^), B (0.52 mg L^−1^), Mn (0.49 mg L^−1^), Zn (0.05 mg L^−1^), Cu (0.01 mg L^−1^), Mo (0.01 mg L^−1^), and S (32.1 mg L^−1^) were supplied. To adjust concentration of nutrient solution, A solution (KNO_3_, 5[Ca(NO_3_)_2_·2H_2_O]NH_4_NO_3_, NH_4_NO_3_, and EDTA-Fe) and B solution (KNO_3_, KH_2_PO_4_, MgSO_4_·7H_2_O, H_3_BO_3_, MnSO_4_·7H_2_O, ZnSO_4_·7H_2_O, CuSO_4_·5H_2_O, and Na_2_MoO_4_·2H_2_O) were mixed. Individual flowers were individually tagged for control fruits and completely covered from light by black colored high-density polyethylene bags for a fruit covering in August 2017 (first trial) and January 2018 (second trial). The control (non-covered) and covered fruit was harvested on October 2017 (first trial) and March 2018 (second trial). Average outside temperatures during fruit covering were 22 °C and 5 °C for the first and second trials, respectively, and temperatures inside the greenhouse were maintained at 25 °C and 18 °C. The average quantity of light (photosynthetically active radiation, PAR) was 336 and 139 (μmol m^−2^ s^−1^) during the first and second trial, respectively. The maximum PAR was 2105 and 904 μmol m^−2^ s^−1^ during the first and second trial, respectively. At least three fruits per developmental stage were harvested within 50–80 days post-anthesis (DPA), and the pericarps were immediately frozen using liquid nitrogen. All samples were stored at −80 °C. 

### 2.2. RNA Isolation and Gene Expression Analysis

The pericarps were ground to fine powder using liquid nitrogen. Total RNA was isolated from 100 mg of pericarp powder using Ribospin™ Seed/Fruit (GeneAll, Seoul, Korea), and 1 μg of total RNA with an 18 bp oligo dT primer was reverse-transcribed to synthesize first-strand cDNA using the DiaStar™ RT Kit (Solgent, Daejeon, Korea) according to the manufacturer’s instructions. Quantitative real-time PCR (qRT-PCR) was conducted using the *Power* SYBR^®^ Green PCR Master Mix (Applied Biosystems, Waltham, MA, USA) according to the manufacturer’s instructions with gene-specific primers (Table S1) and 100 ng of cDNA. At least three biological replications and two technical replications were used. *Actin* for tomato (Solyc03g078400.3) and *ubiquitin* (LOC107854152) for pepper were used as reference genes to normalize gene expression. The amplification program consisted of initial denaturation at 95 °C for 10 min, followed by 40 subsequent cycles of denaturation at 95 °C for 15 s, and annealing and extension at 60 °C for 1 min. qRT-PCR was carried out on a StepOneplus™ Thermal Cycler (Applied Biosystems, Foster City, CA, USA) and analyzed using StepOne v2.3 software (Applied Biosystems, Waltham, MA, USA). Following the reactions, the specificity of the PCR amplification was assessed using melting curve analysis. To normalize the expression levels, the transcription level of tomato actin (Solyc03g078400) and pepper ubiquitin (XM_016699146) was used as a control and the expression level was analyzed as previously described [23]. The primer efficiency was calculated using a 1:10 serial dilution standard curve containing four points and three technical replicates.

### 2.3. Extraction of Carotenoid and Chlorophyll and HPLC Analysis

Carotenoid and chlorophyll extraction and high-performance liquid chromatography (HPLC, 1260 Infinity Series, Agilent, Santa Clara, CA, USA) were conducted as described previously [24]. About 100 mg of frozen pericarp powder was homogenized for 1 min using a FastPrep-24™ instrument (MP Biomedicals, Santa Ana, CA, USA) with two 6 mm glass beads, 15 mg of Mg-carbonate, 300 μL of tetrahydrofuran (THF), and 300 μL of methanol (MeOH) containing 5% butylated hydroxyl-toluene (BHT). Carotenoid and chlorophyll extracts were transferred to a Spin-X centrifuge filter tube (0.45 mm nylon filter, Corning Incorporated, Corning, NY, USA) and centrifuged for 1 min at 1724 g and 4 °C. The filtered extract was transferred into a new 2 mL tube and the debris incubated for 15 min on ice with 350 μL of THF and subsequently centrifuged for 5 min at 1724 g and 4 °C. Following this, 150 μL of THF was added to the debris and the previous step was repeated. The filtered extract was combined with the previous extract, and 150 μL of 25% sodium chloride and 375 μL of petroleum ether were added. After vortexing, the extract was centrifuged for 3 min and the upper phase was transferred to a new 2 mL tube. For re-extraction, 500 μL of petroleum ether was added and the upper phase separated, as described in the previous step. The petroleum ether extract was dried using a MICRO-CENVAC machine (NB-503CIR, N-BIOTEK, Bucheon, Korea) for 2 h at 45 °C. Saponification was conducted on ripe pepper fruit using 10% KOH dissolved in 100% MeOH, as previously described [25]. Chlorophyll was purchased from Sigma-Aldrich (St. Louis, MO USA), and lutein, zeaxanthin, phytoene, phytofluene, β-carotene, capsanthin, and lycopene were purchased from CaroteNature (Lupsingen, Switzerland) (Figure S1).

### 2.4. Flavonoid Extraction and HPLC/LC-MS/MS Analysis

Naringenin chalcone was extracted from tomato and analyzed using HPLC, as described previously [26]. Briefly, 50 mg of frozen pericarp powder was extracted with 1.5 mL of MeOH and sonicated for 15 min. After centrifugation for 10 min at 3000 g, the supernatant was filtered through a 0.22 μm syringe filter (SmartPor^®^-II NYLON syringe filter, 13 mm/0.22 μm, Woongki Science Co., Ltd., Seoul, Korea). Filtered extracts were collected in 2 mL vials and immediately analyzed using HPLC (Shimadzu, Prominence, Kyoto, Japan) with a C_18_ column (4.6 × 250 mm, 5 μm, Waters, Milford, MA, USA) at the Kyungpook National University Instrumental Analysis Center (Daegu, Korea). The column temperature was maintained at 40 °C. The injection volume was 25 μL and the flow rate was 0.7 mL min^−1^. Acetonitrile with 0.1% trifluoroacetic acid (A) and water (B) was used as mobile phase under the following conditions: 5–25% A (0–30 min); 25–30% A (30–35 min); 30–50% A (35–37 min); 50–50% A (37–40 min); and 50–5% A (40–45 min). 

Flavonoids from pepper pericarps were extracted as previously described [2]. Briefly, 500 mg of frozen pericarp powder was extracted in a 2-mL screw-cap tube with 1 mL of MeOH and immediately homogenized for 1 min. Homogenized samples were sonicated for 10 min at room temperature and incubated for 3 h under shaking at room temperature. After incubation, extracts were centrifuged for 10 min at 18,214 g, and the supernatant was moved to a new 1.5 mL tube. Following this, 0.6 mL of 3 M HCl was added and incubated for 90 min at 90 °C and cooled to room temperature. Flavonoid extracts were filtered through a 0.22-μm syringe filter and analyzed with a tandem mass spectrometry system using ESI coupled with HPLC (ACQUITY UPLC H-Class Core System, Waters, Milford, MA, USA) with a C_18_ column (2.1 × 100 mm, 2.5 μm, Waters, Milford, MA, USA). The injection volume was 3 μL and the flow rate was 0.4 mL min^−1^. The column temperature was maintained at 40 °C. The flavonoids were separated using a mobile phase consisting of water with 0.1% formic acid (A) and acetonitrile (B) under the following conditions: 5% B (0–0.5 min); 5–100% B (0.5–3 min); 100–100% B (3–5 min); 100–5% B (5–5.1 min); and 5–5% B (5.1–7.5 min). For ESI-MS (Xevo TQ-S micro, Waters, Milford, MA, USA), the ionization of flavonoids was conducted at a desolvation temperature of 250 °C. The capillary voltage was 3 kV. Naringenin chalcone, quercetin, luteolin, and apigenin were purchased from Sigma-Aldrich (St. Louis, MO, USA).

### 2.5. Volatile Extraction and GC-MS Analysis 

Volatile extraction and analysis were conducted as described previously [27]. First, 3 g of frozen pericarps were collected in a 10 mL headspace glass vials and incubated at 37 °C for 10 min in a water bath. Then, 3 mL of 100 mM EDTA (pH 7.5) and 6.6 g of CaCl_2_·2H_2_O were added and sonicated for 5 min. Volatile extracts were analyzed within 12 h. The samples in the 10-mL headspace glass vials were pre-heated for 10 min at 50 °C. Volatiles were extracted by exposing 65 μm polydimethylsiloxane-divinylbenzene fiber (Supelco, Inc., Bellefonte, PA, USA) to the vial headspace for 10 min at 50 °C. Volatile analysis was conducted on a gas chromatograph-mass spectrometer (7890B-5977B GC/MSD, Agilent, Santa Clara, CA, USA) at the Kyungpook National University Instrumental Analysis Center (Daegu, Korea). The volatiles were desorbed in the injection port of the gas chromatograph for 1 min at 250 °C in splitless mode. Separation was performed on a DB-5 ms column (60 m × 0.25 mm, 1-μm film thickness; J&W Scientific, Folsom, CA, USA). Helium was used as the carrier gas at a flow rate of 1.2 mL min^−1^. The temperature program started at 35 °C for 2 min, followed by a 5 °C min^−1^ ramp to 250 °C, with a 5 min hold at 250 °C. Mass spectra were obtained at an ionization energy of 70 eV and a scan speed of 7 scans s^−1^, with a mass-to-charge ratio scan range of 35 to 220. Volatiles were tentatively identified by comparison (match > 80%) between the experimental MS spectra and those from the mass spectral library of the Wiley Registry (11th Edition/NIST 2017). To validate the reliability of the volatile identification, retention time and MS spectra of β-ionone (Sigma-Aldrich, St. Louis, MO, USA) and geranylacetone (Tokyo Chemical industry Co. Ltd., Tokyo, Japan) were compared with the experimental data. Chromatograms were analyzed using MSD ChemStation Data Analysis software (Agilent, Santa Clara, CA, USA). 

### 2.6. Statistical Analysis

Statistical analysis was conducted using SPSS 25 software (IBM, Armonk, NY, USA) on pigment and flavor levels and the relative expression of carotenoid and flavonoid biosynthetic genes in tomatoes and peppers. Independent *t*-tests were employed to determine significant differences at a level of 5% and 1%.

## 3. Results

### 3.1. Fruit Pigmentation Under the Fruit Covering

In order to gain insight into light-dependent pigmentation in Solanaceae fruits, individual flowers of tomato and pepper were covered with high-density polyethylene bags until fruit harvest (50–80 d). Overall, in the second trial, control fruits (non-covered fruits) harvested in winter exhibited delayed ripening compared to the first trial harvested in summer, presumably due to the lower temperatures during winter.

**Figure 1:**
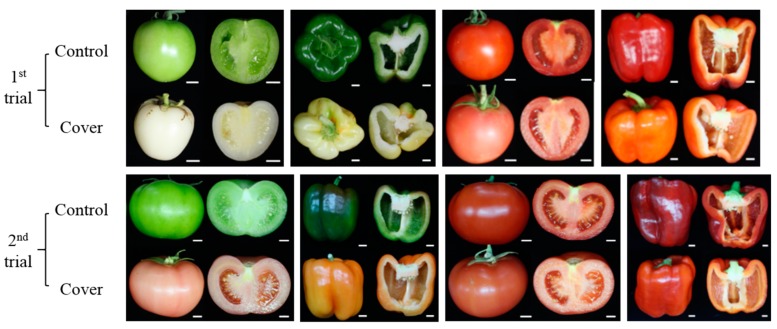
Tomato and pepper fruits under normal and cover conditions. Immature tomato and pepper fruit were covered with high-density polyethylene bags for 50–80 days and harvested on October 2017 (first trial) and March 2018 (second trial). Bar = 1 cm.

The immature control tomatoes in the first and second trial had a typical green color, while the immature covered tomatoes were white and light pink in the first and second trial, respectively (Figure 1). The immature control peppers in both trials were green, while the covered peppers were ivory in the first trial and yellow in the second trial. In the second trial, the light pink and yellow colors of the tomatoes and peppers indicate that the initiation of pigmentation was promoted by the light deficiency for both plants. In both trials, the ripe tomatoes and peppers were a lighter red under fruit covering, compared to the deeper red control fruit (Figure 1). 

In all trials, size and shape of the control and covered fruits were similar in both plants, suggesting that the light deficiency during fruit development and ripening may not influence fruit morphology since a fruit is a sink organ. In addition, fruit pigmentation was even under the fruit covering, although the levels of pigments were decreased. These results indicate that light may not be a limiting factor for fruit pigmentation, but it positively affects fruit pigmentation in tomato and pepper.

### 3.2. Regulation of Pigment Accumulation in Immature Fruit by the Fruit Covering 

The chlorophyll and carotenoids in the control and covered fruit were analyzed using HPLC, and flavonoids were analyzed using liquid chromatography coupled with tandem mass spectrometry (LC-MS/MS). Since ripening was delayed in the second trial (Figure 1), chlorophyll and carotenoid levels were lower than in the first trial. In control fruits, total chlorophylls were accumulated at high amounts in the first and second trial in both plants (Table 1, Figure S2). In both fruits, chlorophyll was not detected in the immature fruits under fruit covering in either trial. Light is essential for chlorophyll synthesis and chloroplast differentiation, and the expression of biosynthetic genes for chlorophyll is mediated by phytochrome under light conditions [9]. Therefore, the absence of chlorophyll in immature fruit under fruit covering may be caused by defective chloroplast development and the suppression of biosynthetic gene expression for chlorophyll.

There were some differences in carotenoid and flavonoid accumulation between the control and covered immature tomatoes. In both trials, lutein and β-carotene accumulated in immature control tomato fruit, while *trans*-lycopene accumulated only in the covered tomatoes (Table 1). In the second trial, phytoene, phytofluene, γ-carotene, and *cis*-lycopene accumulated in the covered fruit, with total carotenoid levels in the covered fruit (37.60 ± 3.35 μg g^−1^ FW) being eight-fold higher than in the control fruit (4.66 ± 0.24 μg g^−1^ FW). The naringenin chalcone, a dominant flavonoid in ripe tomatoes, accumulated in immature covered fruit in the second trial, in contrast to the control fruit (Table 2). *trans*-Lycopene, phytoene, phytofluene, and naringenin chalcone were mainly accumulated in ripe tomato fruit [6,28]. These compounds accumulated in immature tomatoes under fruit covering, but not in the control, indicating a deficiency of light or that fruit pigmentation was indicated earlier in the fruit covering. Even though we did not observe clear early pigmentation under fruit covering in the first trial compared to the second trial, *trans*-lycopene was detected in the covered fruit. We expect to observe visual early pigmentation when the duration of fruit covering in the first is extended. A previous study found that a tomato *high pigment 1* (*hp1*) mutation at the *UV-damaged DNA binding protein 1* (*DDB1*) locus, which is a negative regulator of light signal transduction, delayed ripening by about 4.7 days and delayed ethylene production via the lower expression of the ACC oxidase genes *ACO1*, *ACO3*, and *ACO6* compared to the wild-type [29], while chloroplast degradation and lycopene accumulation was accelerated under shaded conditions in red grapefruit [30]. As a result, it can be concluded that the deficiency of light promotes fruit ripening.

In the first trial, carotenoids did not accumulate in the covered immature peppers, whereas lutein and β-carotene accumulated in the control pepper fruit (Table 1). Capsanthin levels in the covered immature peppers (7.68 ± 3.28 μg g^−1^ FW) were two-fold higher than in the control fruit (3.24 ± 0.70 μg g^−1^ FW), even though total carotenoid levels under fruit covering were 1.6-times lower than in the control fruit in the second trial. These results indicate that the coloration of peppers was promoted under fruit covering by light avoidance. Levels of luteolin were more than 30-times lower in the covered fruits in both trials (Table 2), while quercetin and apigenin levels decreased more than 3-fold and 1.6-fold, respectively. This is in agreement with lower flavonoid accumulation observed in young grapes (*Vitis vinifera* cv. *Cabernet Sauvignon*) kept in the dark for 14 days following bagging with a waterproof box [31]. Flavonoid levels slightly recovered when the grapes were subsequently exposed to UV light, indicating that UV light is required to produce flavonoids, especially flavonols. 

### 3.3. Relative Expression of Carotenoid Biosynthetic Genes in Immature Fruit

**Figure 2:**
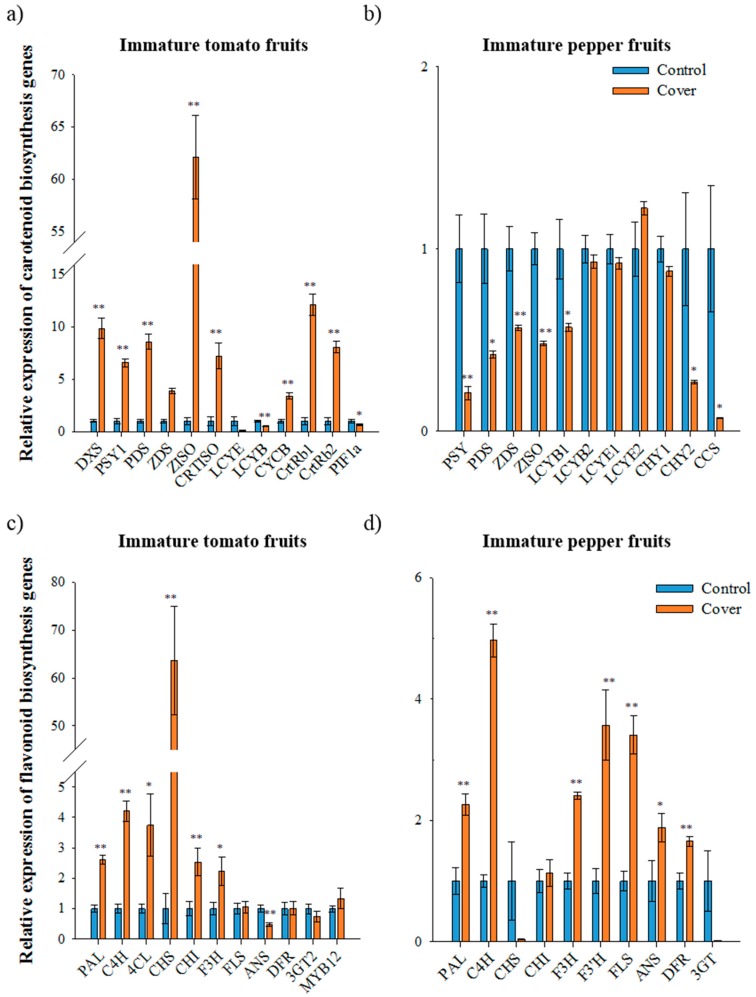
Relative expression of carotenoid and flavonoid biosynthetic genes in immature fruits under normal and cover conditions analyzed using qRT-PCR. The expression of carotenoid biosynthetic genes in immature tomato fruit (second trial) (**a**) and immature pepper fruit (first trial) (**b**). The expression of flavonoid biosynthetic genes in immature tomato fruit (second trial) (**c**) and immature pepper fruit (first trial) (**d**). The data represents the mean of at least three biological replicates and the error bars indicate the standard error (SE). The asterisk * and ** indicate a significant difference at *p* < 0.05 and *p* < 0.01, respectively, according to independent *t*-tests. DXS, deoxyxylulose 5-phosphate synthase; PSY1, phytoene synthase1; PDS, phytoene desaturase; ZDS, ζ-carotene desaturase; ZISO, ζ-carotene isomerase; CRTISO, carotene isomerase; LCYE, lycopene ε-cyclase; LCYB, lycopene β-cyclase; CYCB, chromoplast specific lycopene β-cyclase; CrtRb and CHY, β-carotene hydroxylase; PIF1a, phytochrome-interacting factor1; CCS, capsanthin-capsorubin synthase. PAL, phenylalanine ammonia lyase; C4H, cinnamate 4-hydroxylase; 4CL, 4-coumaroyl CoA ligase; CHS, chalcone synthase; CHI, CHALCONE ISOMERASE; F3H, flavanone 3-hydroxylase; F3′H, flavonoid-3′-hydroxylase; FLS, flavonol synthase; ANS, anthocyanidin synthase; DFR, dihydroflavonol-4-reductase; 3GT, flavonoid 3-O-glucosyltransferase; MYB12, SlR2R3-MYB.

To determine whether changes in pigment accumulation in immature tomato and pepper fruits under light deficiency condition were associated with expression of carotenoid and flavonoid biosynthetic genes, their transcripts were analyzed using qRT-PCR in tomatoes (2nd trial samples) and peppers (1st trial samples). In immature tomato fruits, the expression of the genes for carotenoid biosynthesis—*deoxyxylulose 5-phosphate synthase* (*DXS*), *PSY1*, *phytoene desaturase* (*PDS*), *ζ-carotene desaturase* (*ZDS*), *ζ-carotene isomerase* (*ZISO*), *carotene isomerase* (*CRTISO*), *chromoplast specific lycopene β-cyclase* (*CYCB*), *β-carotene hydroxylase 1* and *2* (*CrtRb1* and *2*)—was higher in the covered fruits than in the control fruits (Figure 2A). In particular, *ZISO* and *PSY* expression in covered fruits was 62- and 6-times higher than in the control fruits, respectively. In contrast, *lycopene ε-cyclase* (*LCYE*) and *lycopene β-cyclase* (*LCYB*) expression under fruit covering was 13- and 2-times lower than in the control, respectively. These results suggested that light deficiency presumably induces the upregulation of carotenoid biosynthetic genes and subsequently carotenoids (especially *trans*-lycopene) accumulated earlier in covered immature tomato fruits than in the control fruit. Additionally, the upregulation of genes operating upstream of lycopene and the downregulation of genes operating downstream of lycopene may positively affect the accumulation of *trans*-lycopene in immature tomato fruit under fruit covering [6].

In immature pepper fruits, expression of *PSY*, *PDS*, *ZDS*, *ZISO*, *LCYB1*, *CHY2*, and *CCS* was significantly downregulated under fruit covering (Figure 2B). In particular, the expression of *PSY* and *PDS* under light deficiency condition was about 5-fold and 2-fold lower than the control. The expression of *CCS* in covered immature pepper fruits was 14-fold lower than in control fruits. This result is consistent with the finding that the carotenoid levels of pepper leaves are lower in the absence of light due to the downregulation of *PSY* and *PDS* [32]. 

### 3.4. Relative Expression of Flavonoid Biosynthetic Genes in Immature Fruit

The expression of the flavonoid biosynthetic genes phenylalanine ammonia lyase (PAL), cinnamate 4-hydroxylase (C4H), 4-coumaroyl coA ligase (4CL), CHS, chalcone isomerase (CHI), and flavanone 3-hydroxylase (F3H) was significantly higher in covered immature tomatoes than in the control (second trial samples, Figure 2C). In particular, CHS expression was 64-times higher in covered immature tomatoes than in the control fruits. The expression of MYB12, a transcription factor responsible for the accumulation of naringenin chalcone [28], exhibited a 1.3-fold increase in covered fruits, while transcript levels were significantly lower for anthocyanidin synthase (ANS) and slightly lower for flavonoid 3-o-glucosyltransferase (3GT) in covered fruits. Expression levels of FLS and dihydroflavonol-4-reductase (DFR) was not altered in the control and covered fruit. The tomatoes started ripening earlier as compared to under the light; these results suggest that in light deficiency conditions, the upregulation of MYB12 and CHS seems to propel the accumulation of naringenin chalcone in immature tomato fruits. 

In covered immature pepper fruits, the expression of flavonoid biosynthetic genes, including *PAL*, *C4H*, *F3H*, *F3′H*, *FLS*, *ANS*, and *DFR,* was significantly higher and expression of *CHI* was slightly higher than in the control fruits (first trial samples, Figure 2D). The *CHS* and *3GT* transcript levels in covered immature peppers were 26 and 71 times lower than that of the control fruits, respectively. Even though many genes regulating flavonoid biosynthesis were upregulated due to a deficiency in light, total flavonoid levels in covered fruits were 32-fold lower than in the control fruits. Therefore, two genes, *CHS* and *3GT*, downregulated under light deficiency, are expected to be an important factor controlling flavonoid biosynthesis. Since *3GT* convert anthocyanidin to anthocyanin in flavonoid biosynthetic pathways, it may not be a limiting factor, especially in relation to accumulation of flavonoids in pepper fruits. These results suggest that *CHS* is the most important factor for accumulation of flavonoid in immature peppers fruits.

### 3.5. Regulation of Pigment Accumulation in Ripe Fruit by Fruit Covering

Seven carotenoids were detected in ripe tomato fruits regardless of light conditions, although the levels of total carotenoids and *trans*-lycopene in covered tomatoes were 50% lower than that of the control in both trials (Table 1, Figure S3), as previously reported [12]. In covered ripe tomatoes, naringenin chalcone levels were 11- and 93-times lower in the first and second trials, respectively, than the control (Table 2). In covered ripe peppers, total carotenoids and capsanthin content was 25% lower than the control in both trials (Table 1, Figures S2 and S3). Luteolin and quercetin levels were significantly lower in covered pepper fruits compared to the control. Apigenin content in covered pepper fruits were 3- and 1.2-fold lower in the first and second trail, respectively, than in the control. In addition, total flavonoid levels in the covered ripe peppers were 18- and 7-fold lower in the first and second trials than in the control peppers (Table 2). 

We cannot reject the possibility that fruit covering is governed by other environmental factors, including temperature and humidity, which were not monitored, even though slight increases were expected. Although high temperatures (30–50 °C) can reduce lycopene levels, β-carotene levels are not affected by high temperatures in tomato [33]. However, levels of β-carotene were significantly decreased under cover in both tomato and pepper fruits (Table 1). The reduced level of β-carotene under cover was presumably due to light deficiency, and not temperature conditions. 

This result indicates that different amounts of carotenoids and β-carotene accumulation were presumably regulated by different light conditions in the control and covered fruits. Light is known to be a main driver for accumulation of flavonoids, which is higher in sun-exposed grapes and apples than in dark or shade-grown fruits [15,34,35]. Furthermore, flavonoid accumulation is positively influenced by light in both climacteric and non-climacteric fruit [11]. In the present study, flavonoid levels were lower in both tomato (climacteric) and pepper (non-climacteric) under cover, suggesting that light can independently control flavonoid biosynthesis, regardless of the ripening mechanism.

### 3.6. Relative Expression of Carotenoid Biosynthetic Genes in Ripe Fruit

**Figure 3:**
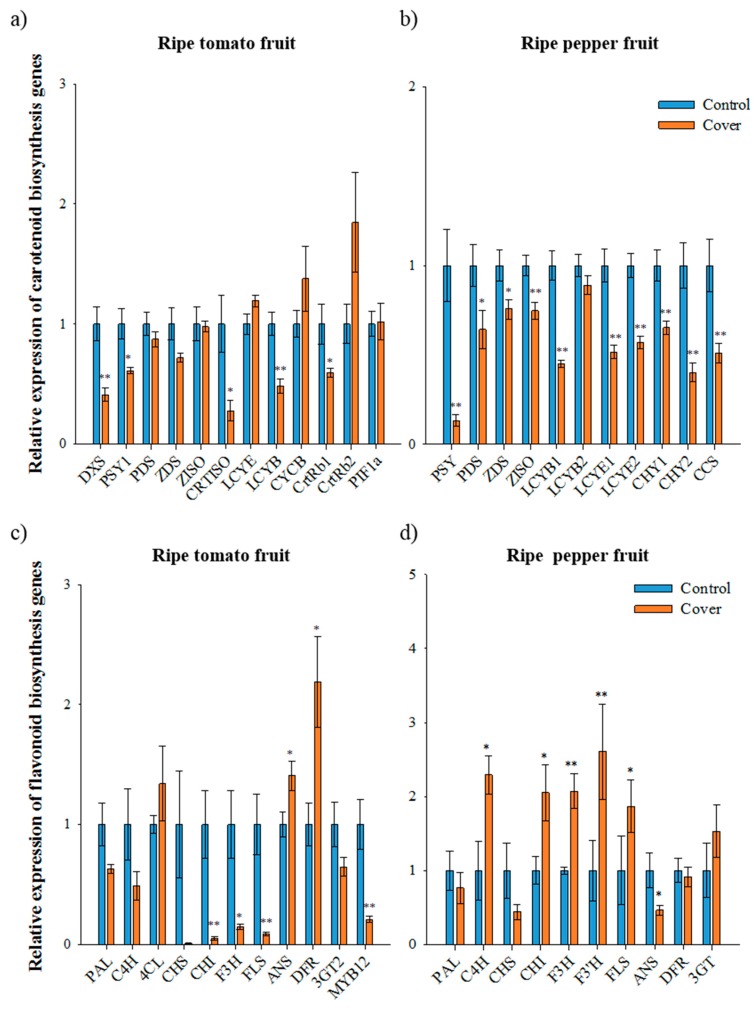
Relative expression of carotenoid and flavonoid biosynthetic genes in ripe fruits under normal and cover conditions analyzed using qRT-PCR. The expression of carotenoid biosynthetic genes in ripe tomato fruit (second trial) (**a**) and ripe pepper fruit (first trial) (**b**). The expression of flavonoid biosynthetic genes in ripe tomato fruit (second trial) (**c**) and ripe pepper fruit (first trial) (**d**). Data represents the mean of at least three biological replicates and the error bars indicate standard error (SE). The asterisk * and ** indicate a significant difference at *p* < 0.05 and *p* < 0.01, respectively, according to an independent *t*-test.

In contrast to immature fruit, the expression of most carotenoid biosynthetic genes in covered ripe tomatoes was lower than that in the control tomatoes, with the exception of *LCYE*, *CYCB*, and *CrtRb2* (second trial samples, Figure 3A). In the covered tomato fruit, *PSY1* and *CRTISO* expression was 1.6-fold and 3.6-fold lower than in the control, respectively. The expression of *PIF1a*, a repressor of *PSY1* [14], was not significantly altered under a light deficiency condition in ripe tomato fruits; thus, the lower expression of *PSY1* in covered fruits was presumably indirectly regulated by *PIF1a*. In carotenoid biosynthesis, the reduced expression of upstream genes, especially *PSY1* and *CRTISO*, is closely associated with the lower accumulation of lycopene and total carotenoids in covered tomato fruit. In addition, the higher expression of *CYCB* and *CrtRb* in covered tomato fruit during the ripening progress can reduce lycopene accumulation [6]. The manipulation of the tomato genes involved in light signaling has been shown to affect fruit pigmentation in tomatoes [9,36]. The downregulation of the photomorphogenic transcription factor *SlHY5* results in thylakoid-deficient chloroplasts with larger plastoglobules in the green stages and lower carotenoid levels in ripe fruit [36]. In addition, the silencing of the negative regulators of light-signaling *SlDDB1* and *SlCUL4* led to a significant increase in the number of plastids, resulting in enhanced carotenoid and flavonoid accumulation during ripening [37]. In particular, the positive role of the PHY-dependent light response cascade in fruit carotenogenesis has been demonstrated [13,14].

In ripe pepper fruit, the expression of all carotenoid biosynthetic genes was significantly lower in covered fruits than in the control (first trial samples, Figure 3B). The expression of *PSY* and *CCS* in covered fruits was 8-fold and 2-fold lower than in the control, respectively, while the expression of *LCYB1*, *CHY1*, and *CHY2* in covered fruits decreased 2-, 1.5-, and 2.5-fold, respectively. Total carotenoids and capsanthin accumulation has been found to be correlated with the transcript levels of carotenoid biosynthetic genes in pepper under different light conditions. For a higher accumulation of carotenoids in pepper, a greater expression of *PSY*, *PDS*, *CHY*, and *CCS* and minimal expression of *LCYB* during fruit ripening is required [7]. Therefore, the reduced expression of all carotenoid biosynthetic genes was involved in lower carotenoid levels in the covered pepper fruits. Since carotenoid hydroxylation is necessary for capsanthin and capsorubin biosynthesis in pepper, light may be an important driver for the control, unlike tomato. In the case of tomato, xanthophylls are rarely found in ripe tomatoes.

### 3.7. Relative Expression of Flavonoid Biosynthetic Genes in Ripe Fruit 

Expression of the flavonoid biosynthetic genes *CHS*, *CHI*, *F3H, FLS*, and *MYB12* in ripe tomatoes was significantly lower under a light deficiency condition than that of the control (second trial samples, Figure 3C). In particular, the expression of *CHS* in the covered fruits was 111.11-times lower than that of the control. Furthermore, the expression of *MYB12* in the covered fruits was 5-times lower than that in the control. The expression of *4CL*, *ANS,* and *DFR* was higher in the covered tomatoes than those in the control and expression of *PAL*, *C4H*, and *3GT* was not altered under different light conditions. These results are consistent with the finding that the expression of *CHS* and *MYB12* is correlated with the concentration of naringenin chalcone in tomato fruit [28]. In the covered ripe peppers, the expression of *C4H*, *CHI*, *F3H*, *F3′H*, and *FLS* was higher than that in the control (first trial samples, Figure 3D). The expression of *CHS* and *ANS* in the covered fruits was 2-fold lower than that in the control. Although the expression of several flavonoid biosynthetic genes was higher in covered pepper fruits, flavonoid levels were significantly lower than that in the control. The expression of *CHS* was consistent with levels of flavonoids in both immature and ripe fruit, suggesting that *CHS* may act as an important factor for flavonoid accumulation in pepper fruits.

The changes in expression of *CHI*, *F3H*, and *FLS* in ripe tomato and pepper fruits are completely different—a decrease in tomato and increase in pepper under light deficiency (Figure 3C,D). In covered ripe fruits, *CHS* expression was found to be decreased for both plants. Although the major compounds differed between ripe tomatoes and peppers, *CHS* seem to be a key regulator controlling flavonoid accumulation in both fruits, and *SlMYB12* may serve the accumulation of naringenin chalcone in ripe tomatoes. Furthermore, the increased expression of flavonoid biosynthetic genes in covered fruit did not positively affect the accumulation of flavonoid in peppers fruits. The promoter region of *CHS* has light regulatory units consisting of MYB-recognition (MRE) and ACGT-containing elements (ACE) [38]. These units respond to light and induce the expression of *CHS* via transcription factors such as R2R3-MYB and bHLH. Therefore, covering of fruit may interrupt the light response in inducing expression of *CHS*, and flavonoid levels could subsequently be repressed in both tomato and pepper fruit.

### 3.8. Flavor Volatile Analysis of Tomato and Pepper Fruit Under Cover

Volatile compounds from ripe tomato and peppers were captured using solid phase microextraction (SPME) and analyzed using gas chromatography-mass spectrometry (GC-MS). A total of 38 and 31 volatile compounds were detected in ripe tomato and pepper fruits, respectively (Table S2, Figure S4). Of these, 25 are considered to be flavor volatiles [20,39], and were divided into eight groups according to their change patterns under the conditions of light avoidance and importance for flavor (Table 3). Group 1 showed a similar change pattern in the cover/control ratio for tomatoes and peppers, while Group 2 exhibited a different pattern. Group 3 was characterized by similar concentrations in the control and covered fruits in both tomatoes and peppers. Group 4 and Group 5 had lower cover/control ratios in tomatoes and peppers, respectively, while Group 6 had a higher cover/control ratio in peppers. Finally, Group 7 and Group 8 were detected only in tomatoes and peppers, respectively. Seven compounds belonging to Group 1 exhibited light-dependent regulation in both tomatoes and peppers fruits, four of which were found to be increased in the covered fruits: (*E*)-oct-2-enal, (*2E*,*4E*)-deca-2,4-dienal, (*E*)-hept-2-enal, and 2-pentylfuran. In contrast, (*E*)-hex-2-enal was lower in the covered fruits compared to the control. In both ripe tomatoes and peppers fruits, linalool was detected only in the control, while (*2E*,*4E*)-nona-2,4-dienal was detected only in the covered fruits. 

More than 400 volatiles were detected in tomatoes, but only 15–20 were found to have an impact on flavor in sufficient quantities [20]. We identified 13 compounds involved with the flavor of tomatoes. Among them, four compounds (2-(2-methylpropyl)-1,3-thiazol, 2-phenylacetaldehyde, 2-phenylethanol, and 2-nitroethylbenzene) were detected only in the non-covered control fruit. Three compounds ((*E*)-hept-2-enal, beta-damascenone, and geranylacetone) were found increased in the covered fruits than in the control. Of the 13 volatiles, four volatiles (geranylacetone, beta-damascenone, *trans*-beta-ionone, and 6-methyl-5-hepten-2-one) were derived from carotenoids. In tomatoes, the levels of all carotenoids were lower in the covered fruits, but geranylacetone and beta-damascenone were higher in the covered fruits, compared to the control. These results indicate that carotenoid catabolic genes/proteins, for example, carotenoid cleavage dioxygenase, may be regulated by light; however, further research is necessary. In previous research, geranylacetone was reported to be related to flavor and sweetness in tomatoes, while the intensity of sweetness was related to hexanal, *cis*-3-hexenal, 2-hexenal, or *cis*-3-hexenol [40]. Geranylacetone was found to be higher in the covered tomatoes compared to the control, while hexanal content, (*Z*)-hex-3-en-1-ol and (*E*)-hex-2-enal, were found to be lower in covered fruits. Flavor volatiles in pepper have not been greatly studied in the past. We identified four compounds that act as flavor in peppers [41]. Of these, two were detected only in the covered peppers ((*2E*,*4Z*)-deca-2,4-dienal and (*2E*,*4E*)-nona-2,4-dienal) and two were slightly increased in covered pepper fruits (hexanal and 2-methoxy-3-(2-methylpropyl)pyrazine). The odor of the increased compounds in covered tomato and pepper fruits was predominantly described as green/leaf [39], indicating that the synthesis of these volatiles in tomato and pepper fruit is upregulated or prolonged under light deficiency conditions. These results suggest that under different light conditions, changes in flavor volatiles can be made to improve fruit flavor.

## 4. Conclusions

Little is known about light-controlled fruit pigmentation and flavor volatiles in tomato and pepper fruit. In this study, we examined the effect of light on levels of pigments and flavor volatiles in the two fruits. We also monitored the expression of structural genes and transcription factors on pigment accumulation. Overall, the accumulation of carotenoids and flavonoids in both tomato and peppers was affected by light conditions; it was found to be lower with light deficiency, regardless of the developmental stage in the two trials. Expression analysis revealed that the transcript levels of *PSY* and *CHS* were the primary determinant of the accumulation of carotenoids and flavonoids, respectively, in response to light in both tomatoes and peppers. Pigmentation in the covered fruits was found to be indicated earlier than that in tomato and pepper exposed to light. In terms of volatile emissions, 38 and 31 compounds were detected in tomatoes and peppers, respectively. Of these, seven exhibited similar patterns of change in the cover/control ratio in tomatoes and peppers. Therefore, fruit coverings can modulate specific flavor volatile compounds and enhance fruit quality. We believe that these findings will improve our understanding of the molecular mechanism of health promoting compounds in tomato and pepper fruits.

## Figures and Tables

**Table 1:** Chlorophyll and carotenoid levels (μg g^−1^ FW) in control (non-covered fruits) and covered tomato and pepper fruits. The data represents the mean of at least three biological replicates ± the standard error (SE).

Pigment		Immature Fruits				Ripe Fruits			
		First Trial		Second Trial		First Trial		Second Trial	
Tomato		Control	Cover	Control	Cover	Control	Cover	Control	Cover
Chlorophyll	Chlorophyll a	665.93 ± 28.77	ND	101.31 ± 4.78	ND	ND	ND	ND	ND
	Chlorophyll b	273.67 ± 9.12	ND	69.72 ± 4.59	ND	ND	ND	ND	ND
	Total chlorophyll	939.61 ± 37.84	ND	180.86 ± 27.91	ND	ND	ND	ND	ND
Carotenoid	Phytoene	ND	ND	ND	3.08 ± 0.28 **	32.88 ± 2.18	16.19 ± 1.17 **	6.77 ± 0.84	5.46 ± 0.31
	Phytofluene	ND	ND	ND	1.93 ± 0.18 **	17.95 ± 0.99	7.86 ± 0.65 **	5.11 ± 0.70	4.02 ± 0.23
	Lutein	4.16 ± 0.10	ND	2.56 ± 0.13	ND	ND	ND	1.62 ± 0.05	ND
	β-Carotene	3.68 ± 0.15	ND	2.10 ± 0.13	1.38 ± 0.05 **	8.86 ± 1.00	1.39 ± 0.19 **	8.54 ± 0.71	1.16 ± 0.13 **
	γ-Carotene	ND	ND	ND	5.43 ± 0.52 **	19.85 ± 0.52	8.94 ± 0.60 **	23.33 ± 3.75	9.49 ± 0.47 *
	cis-Lycopene	ND	ND	ND	2.82 ± 0.30 **	8.75 ± 0.86	6.89 ± 0.48	10.98 ± 1.25	3.94 ± 0.48 **
	trans-Lycopene	ND	0.64 ± 0.37	ND	22.95 ± 2.42 **	254.91 ± 26.69	109.31 ± 8.57 **	112.74 ± 23.37	46.33 ± 4.28 *
	Total carotenoid	7.84 ± 0.26	0.64 ± 0.37 **	4.66 ± 0.24	37.60 ± 3.35 **	343.21 ± 27.70	150.59 ± 11.49 **	169.09 ± 29.35	70.41 ± 4.41 *
Pepper		Control	Cover	Control	Cover	Control	Cover	Control	Cover
Chlorophyll	Chlorophyll a	1376.62 ± 59.26	ND	54.64 ± 4.08	ND	ND	ND	ND	ND
	Chlorophyll b	861.21 ± 57.10	ND	196.56 ± 9.18	ND	ND	ND	ND	ND
	Total chlorophyll	2237.84 ± 116.27	ND	251.19 ± 13.22	ND	ND	ND	ND	ND
Carotenoid	Phytoene	ND	ND	ND	ND	ND	ND	0.77 ± 0.09	ND
	Phytofluene	ND	ND	ND	ND	ND	ND	0.71 ± 0.04	ND
	Lutein	9.59 ± 0.38	ND	5.94 ± 0.17	ND	3.94 ± 0.78	0.99 ± 0.10 *	ND	ND
	β-Carotene	4.53 ± 0.23	ND	4.54 ± 0.12	1.32 ± 0.61	5.99 ± 1.75	0.93 ± 0.28 *	13.07 ± 0.99	3.44 ± 0.96 **
	Zeaxanthin	ND	ND	1.48 ± 0.06	1.82 ± 0.29	5.62 ± 1.35	2.19 ± 0.70	6.06 ± 0.72	7.28 ± 1.56
	β-Cryptoxanthin	ND	ND	ND	0.62 ± 0.20	1.70 ± 0.49	0.53 ± 0.14	3.19 ± 0.26	1.44 ± 0.41 **
	Capsanthin	ND	ND	3.24 ± 0.70	7.68 ± 3.28	69.48 ± 15.08	15.66 ± 2.99 *	182.85 ± 7.82	46.09 ± 5.58 **
	Total carotenoid	14.12 ± 0.28	ND	15.20 ± 0.52	9.26 ± 4.06	86.74 ± 19.16	16.69 ± 4.29 *	206.15 ± 9.28	58.25 ± 8.31 **

* and ** indicate a significant difference at *p* < 0.05 and *p* < 0.01, respectively, according to independent *t*-tests. ND: not detected.

**Table 2:** Flavonoid levels (μg g^−1^ FW) in control (non-covered fruits) and covered tomato and pepper fruits. The data represents the mean of at least three biological replicates ± the standard error (SE).

Flavonoid	Immature Fruits				Ripe Fruits			
	First Trial		Second Trial		First Trial		Second Trial	
Tomato	Control	Cover	Control	Cover	Control	Cover	Control	Cover
Naringenin chalcone	ND	ND	ND	13.05 ± 4.06 *	138.16 ± 35.18	12.69 ± 6.78 *	153.54 ± 35.56	1.65 ± 0.78
Pepper	Control	Cover	Control	Cover	Control	Cover	Control	Cover
Luteolin	230.32 ± 32.06	2.07 ± 0.86 **	1.61 ± 1.54	0.05 ± 0.04	107.71 ± 16.68	4.86 ± 1.7 **	259.12 ± 50.59	35.21 ± 10.53 *
Quercetin	13.09 ± 2.05	1.90 ± 0.41 **	5.90 ± 1.45	1.76 ± 1.11	20.18 ± 4.58	3.53 ± 0.53 *	43.26 ± 11.52	4.89 ± 0.77 *
Apigenin	0.85 ± 0.15	0.32 ± 0.21	0.08 ± 0.02	0.05 ± 0.00	1.17 ± 0.31	0.36 ± 0.11	0.66 ± 0.16	0.55 ± 0.12
Total flavonoid	244.26 ± 33.64	4.23 ± 1.17 **	7.59 ± 2.96	1.86 ± 1.07	129.06 ± 16.83	7.36 ± 1.50 **	303.04 ± 61.94	40.66 ± 10.94 *

* and ** indicate a significant difference at *p* < 0.05 and *p* < 0.01, respectively, according to independent *t*-tests. ND: not detected.

**Table 3:** Volatile compounds detected in ripe tomato and pepper fruits under normal and canopy conditions. The data represents the mean of the ratio (canopy/control) for three biological replicates.

Group	R.T.	Volatile Compound	Chemical Class	Odor Description ^a^	Ratio (Cover/Control)	
					Tomato	Pepper
1	15.745	(*E*)-oct-2-enal	Aldehydes	green, leaf, nut	2.40 *	1.53 **
	22.142	*(2E,4E)-deca-2,4-dienal*	Aldehydes	fried, wax, fat	2.29 **	+
	11.854	**(E)-hept-2-enal**	Aldehydes	soap, fat, almond	2.24	2.69
	13.405	2-pentylfuran	Oxygen-containing heterocyclic compounds	green bean, butter	1.50 *	+
	6.984	(*E*)-hex-2-enal	Aldehydes	green, leaf, apple	0.40 *	0.66
	17.011	3,7-dimethylocta-1,6-dien-3-ol (Linalool)	Alcohols	flower, lavender	−	−
	19.909	*(2E,4E)-nona-2,4-dienal*	Aldehydes	green, watermelon	+	+
2	23.552	(*E*)-1-(2,6,6-trimethylcyclohexa-1,3-dien-1-yl)but-2-en-1-one (beta-damascenone)	Carotenoid derivatives	apple, rose, honey	1.84	−
3	14.822	2-ethylhexan-1-ol	Alcohols	rose, green	1.50	1.26
	3.687	**3-methylbutan-1-ol**	Alcohols	whiskey, malt, burnt	1.15	1.24
	19.728	decanal	Aldehydes	soap, orange peel	1.02	1.23
	22.905	2,4-diisocyanato-1-methylbenzene	Nitrogen compounds	sweet, fruity, pungent ^b^	0.90	0.88
	14.117	(*2E*,*4E*)-hepta-2,4-dienal	Aldehydes	nut	0.84	1.21
4	5.195	***hexanal***	Aldehydes	grass	0.43 **	1.40
	19.977	2-(4-methylcyclohex-3-en-1-yl)propanal	Aldehydes	herbal, spice ^c^	−	1.04
5	13.238	**6-methylhept-5-en-2-one (MHO)**	Carotenoid derivatives	fruity, floral ^d^	0.61	−
6	21.669	(*2E*,*4Z*)-deca-2,4-dienal	Aldehydes	fried, fat	1.39	+
7	24.779	**(*5E*)-6,10-dimethylundeca-5,9-dien-2-one (geranylacetone)**	Carotenoid derivatives	fruity, floral ^d^	1.98	ND
	25.443	**(*E*)-4-(2,6,6-trimethylcyclohexen-1-yl)but-3-en-2-one (trans-beta-ionone)**	Carotenoid derivatives	fruity, floral ^d^	0.75	ND
	7.134	**(*Z*)-hex-3-en-1-ol**	Alcohols	grass	0.72	ND
	14.884	**2-(2-methylpropyl)-1,3-thiazole**	Nitrogen compounds	tomato leaf, green	−	ND
	15.243	**2-phenylacetaldehyde**	Aldehydes	honey, sweet	−	ND
	17.349	**2-phenylethanol**	Alcohols	honey, spice, rose, lilac	−	ND
	21.811	**2-nitroethylbenzene**	Nitrogen compounds	flower, spice	−	ND
8	19.138	*2-methoxy-3-(2-methylpropyl)pyrazine*	Pyrazines	earth, spice, green pepper	ND	1.31

+: volatile compounds detected only in canopy-treated fruit, −: volatile compounds detected only in control fruit, ND: not detected, Bold letters: volatile compounds involved in the flavor of tomatoes; italics: volatile compounds involved in the flavor of peppers. Odor descriptions are adapted from ^a^: [39]; ^b^: https://cameochemicals.noaa.gov; ^c^: http://www.thegoodscentscompany.com; ^d^: [20]. * and ** indicate a significant difference at *p* < 0.05 and *p* < 0.01, respectively, according to independent *t*-tests.

## Supplementary Materials

The following are available online at https://www.mdpi.com/2076-3921/9/1/14/s1, Table S1: Primers used in the quantitative real time-PCR of tomato and pepper, Table S2: Volatile compounds detected by GC-MS analysis in tomato and pepper fruits. Figure S1: HPLC chromatogram of analytical standards (chlorophyll, β-carotene, lycopene, capsanthin, phytofluene, and lutein) and carotenoid producing *E. coli* extracts (phytoene, zeaxanthin). Figure S2: HPLC chromatogram of chlorophylls and carotenoids detected at 434 and 471 nm in immature tomato and pepper fruits. Each number indicates that 1, chlorophyll b; 2, lutein; 3, chlorophyll a; 4, β-carotene; 5, trans-lycopene; 6, γ-carotene; 7, cis-lycopene; 8, capsanthin; 9, zeaxanthin; 10, β-cryptoxanthin. Figure S3: HPLC chromatogram of carotenoids detected at 471, 286, and 348 nm in ripe tomato and pepper fruits. Each number indicates that 1, lutein; 2, β-carotene; 3, γ-carotene; 4, cis-lycopene; 5, trans-lycopene; 6, capsanthin; 7, zeaxanthin; 8, β-cryptoxanthin; 9, phytoene; 10, phytofluene. Figure S4: Mass spectrum of volatile compounds listed in Table S2 and analytical standards of β-ionone and geranlyacetone.

## References

[1] Beecher G.R. (1998). Nutrient content of tomatoes and tomato products. Proc. Soc. Exp. Biol. Med..

[2] Bae H., Jayaprakasha G.K., Jifon J., Patil B.S. (2012). Extraction efficiency and validation of an hplc method for flavonoid analysis in peppers. Food Chem..

[3] Nisar N., Li L., Lu S., Khin N.C., Pogson B.J. (2015). Carotenoid metabolism in plants. Mol. Plant.

[4] Heim K.E., Tagliaferro A.R., Bobilya D.J. (2002). Flavonoid antioxidants: Chemistry, metabolism and structure-activity relationships. J. Nutr. Biochem..

[5] Fraser P.D., Bramley P.M. (2004). The biosynthesis and nutritional uses of carotenoids. Prog. Lipid Res..

[6] Liu L., Shao Z., Zhang M., Wang Q. (2015). Regulation of carotenoid metabolism in tomato. Mol. Plant.

[7] Ha S.H., Kim J.B., Park J.S., Lee S.W., Cho K.J. (2007). A comparison of the carotenoid accumulation in capsicum varieties that show different ripening colours: Deletion of the capsanthin-capsorubin synthase gene is not a prerequisite for the formation of a yellow pepper. J. Exp. Bot..

[8] Slimestad R., Fossen T., Verheul M.J. (2008). The flavonoids of tomatoes. J. Agric. Food Chem..

[9] Llorente B., Martinez-Garcia J.F., Stange C., Rodriguez-Concepcion M. (2017). Illuminating colors: Regulation of carotenoid biosynthesis and accumulation by light. Curr. Opin. Plant Biol..

[10] Pizarro L., Stange C. (2009). Light-dependent regulation of carotenoid biosynthesis in plants. Cienc. Investig. Agrar..

[11] Zoratti L., Karppinen K., Luengo Escobar A., Haggman H., Jaakola L. (2014). Light-controlled flavonoid biosynthesis in fruits. Front. Plant Sci..

[12] Raymundo L.C., Chichester C.O., Simpson K.L. (1976). Light-dependent carotenoid synthesis in the tomato fruit. J. Agric. Food Chem..

[13] Cruz A.B., Bianchetti R.E., Alves F.R.R., Purgatto E., Peres L.E.P., Rossi M., Freschi L. (2018). Light, ethylene and auxin signaling interaction regulates carotenoid biosynthesis during tomato fruit ripening. Front. Plant Sci..

[14] Llorente B., D’Andrea L., Ruiz-Sola M.A., Botterweg E., Pulido P., Andilla J., Loza-Alvarez P., Rodriguez-Concepcion M. (2016). Tomato fruit carotenoid biosynthesis is adjusted to actual ripening progression by a light-dependent mechanism. Plant J. Cell Mol. Biol..

[15] Azuma A., Yakushiji H., Koshita Y., Kobayashi S. (2012). Flavonoid biosynthesis-related genes in grape skin are differentially regulated by temperature and light conditions. Planta.

[16] Lu Y., Bu Y., Hao S., Wang Y., Zhang J., Tian J., Yao Y. (2017). Mybs affect the variation in the ratio of anthocyanin and flavanol in fruit peel and flesh in response to shade. J. Photochem. Photobiol. B.

[17] Awad M.A., Wagenmakers P.S., de Jager A. (2001). Effects of light on flavonoid and chlorogenic acid levels in the skin of ‘jonagold’ apples. Sci. Hortic..

[18] Zhang X.D., Allan A.C., Yi Q.O., Chen L.M., Li K.Z., Shu Q., Su J. (2011). Differential gene expression analysis of yunnan red pear, pyrus pyrifolia, during fruit skin coloration. Plant Mol. Biol. Rep..

[19] Tieman D., Bliss P., McIntyre L.M., Blandon-Ubeda A., Bies D., Odabasi A.Z., Rodriguez G.R., van der Knaap E., Taylor M.G., Goulet C. (2012). The chemical interactions underlying tomato flavor preferences. Curr. Biol..

[20] Klee H.J. (2010). Improving the flavor of fresh fruits: Genomics, biochemistry, and biotechnology. New Phytol..

[21] Rosenfeld H.J., Aaby K., Lea P. (2002). Influence of temperature and plant density on sensory quality and volatile terpenoids of carrot (*Daucus carota* L.) root. J. Sci. Food Agric..

[22] Eggink P.M., Maliepaard C., Tikunov Y., Haanstra J.P., Bovy A.G., Visser R.G. (2012). A taste of sweet pepper: Volatile and non-volatile chemical composition of fresh sweet pepper (capsicum annuum) in relation to sensory evaluation of taste. Food Chem..

[23] Schmittgen T.D., Livak K.J. (2008). Analyzing real-time pcr data by the comparative ct method. Nat. Protoc..

[24] Yoo H.J., Park W.J., Lee G.M., Oh C.S., Yeam I., Won D.C., Kim C.K., Lee J.M. (2017). Inferring the genetic determinants of fruit colors in tomato by carotenoid profiling. Molecules.

[25] Kim J.E., Yoo H.J., Kang B.-C., Lee J.M. (2017). A new nonsense mutation in capsanthin/capsorubin synthase controlling orange pepper fruit. Hortic. Sci. Technol..

[26] Bino R.J., Ric de Vos C.H., Lieberman M., Hall R.D., Bovy A., Jonker H.H., Tikunov Y., Lommen A., Moco S., Levin I. (2005). The light-hyperresponsive high pigment-2dg mutation of tomato: Alterations in the fruit metabolome. New Phytol..

[27] Rambla J.L., Medina A., Fernandez-Del-Carmen A., Barrantes W., Grandillo S., Cammareri M., Lopez-Casado G., Rodrigo G., Alonso A., Garcia-Martinez S. (2017). Identification, introgression, and validation of fruit volatile qtls from a red-fruited wild tomato species. J. Exp. Bot..

[28] Adato A., Mandel T., Mintz-Oron S., Venger I., Levy D., Yativ M., Dominguez E., Wang Z., De Vos R.C., Jetter R. (2009). Fruit-surface flavonoid accumulation in tomato is controlled by a slmyb12-regulated transcriptional network. PLoS Genet..

[29] Wang A., Chen D., Ma Q., Rose J.K.C., Fei Z., Liu Y., Giovannoni J.J. (2019). The tomato high pigment1/damaged DNA binding protein 1 gene contributes to regulation of fruit ripening. Hortic. Res..

[30] Lado J., Cronje P., Alquezar B., Page A., Manzi M., Gomez-Cadenas A., Stead A.D., Zacarias L., Rodrigo M.J. (2015). Fruit shading enhances peel color, carotenes accumulation and chromoplast differentiation in red grapefruit. Physiol. Plant..

[31] Koyama K., Ikeda H., Poudel P.R., Goto-Yamamoto N. (2012). Light quality affects flavonoid biosynthesis in young berries of *Cabernet sauvignon* grape. Phytochemistry.

[32] Simkin A.J., Zhu C., Kuntz M., Sandmann G. (2003). Light-dark regulation of carotenoid biosynthesis in pepper (capsicum annuum) leaves. J. Plant Physiol..

[33] Dumas Y., Dadomo M., Di Lucca G., Grolier P. (2003). Effects of environmental factors and agricultural techniques on antioxidantcontent of tomatoes. J. Sci. Food Agric..

[34] Feng F., Li M., Ma F., Cheng L. (2014). Effects of location within the tree canopy on carbohydrates, organic acids, amino acids and phenolic compounds in the fruit peel and flesh from three apple (malus x domestica) cultivars. Hortic. Res..

[35] Blancquaert E.H., Oberholster A., Ricardo-da-Silva J.M., Deloire A.J. (2019). Grape flavonoid evolution and composition under altered light and temperature conditions in *Cabernet sauvignon* (*Vitis vinifera* L.). Front. Plant Sci..

[36] Liu Y., Roof S., Ye Z., Barry C., van Tuinen A., Vrebalov J., Bowler C., Giovannoni J. (2004). Manipulation of light signal transduction as a means of modifying fruit nutritional quality in tomato. Proc. Natl. Acad. Sci. USA.

[37] Wang S., Liu J., Feng Y., Niu X., Giovannoni J., Liu Y. (2008). Altered plastid levels and potential for improved fruit nutrient content by downregulation of the tomato ddb1-interacting protein cul4. Plant. J. Cell Mol. Biol..

[38] Hartmann U., Sagasser M., Mehrtens F., Stracke R., Weisshaar B. (2005). Differential combinatorial interactions of cis-acting elements recognized by r2r3-myb, bzip, and bhlh factors control light-responsive and tissue-specific activation of phenylpropanoid biosynthesis genes. Plant Mol. Biol..

[39] Wang L., Baldwin E., Luo W., Zhao W., Brecht J., Bai J. (2019). Key tomato volatile compounds during postharvest ripening in response to chilling and pre-chilling heat treatments. Postharvest Biol. Technol..

[40] Baldwin E.A., Scott J.W., Shewmaker C.K., Schuch W. (2000). Flavor trivia and tomato aroma: Biochemistry and possible mechanisms for control of important aroma components. Hortscience.

[41] Luning P.A., Derijk T., Wichers H.J., Roozen J.P. (1994). Gas-chromatography, mass-spectrometry, and sniffing port analyses of volatile compounds of fresh bell peppers (capsicum-annuum) at different ripening stages. J. Agric. Food Chem..

