# Supplementary material for: Light-Controlled Fruit Pigmentation and Flavor Volatiles in Tomato and Bell Pepper
Source: Antioxidants (Basel). 2019 Dec 23;9(1):14. doi: 10.3390/antiox9010014 (PMC7023227; doi:10.3390/antiox9010014)
Supplement: Supplementary file 1 [file antioxidants-09-00014-s001.zip › antioxidants-660391-supp-for publish/antioxidants-660391-supp figures-for publish.docx]

**Supplementary figures**


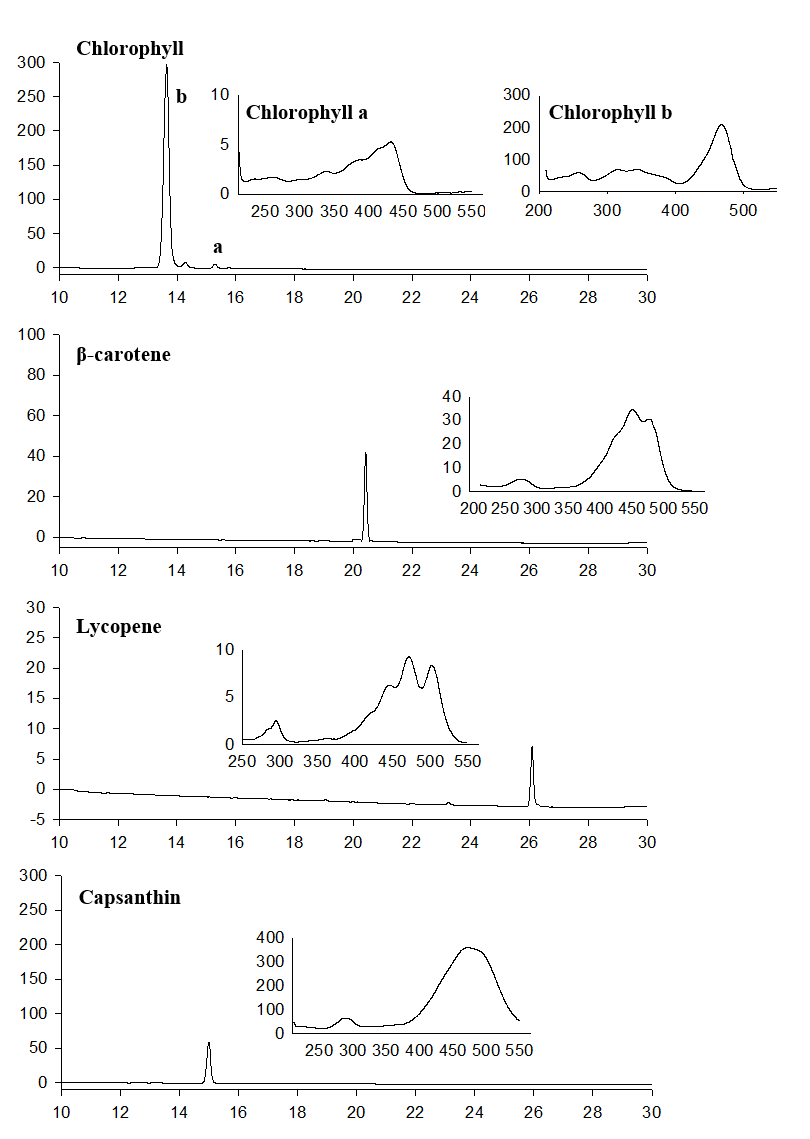

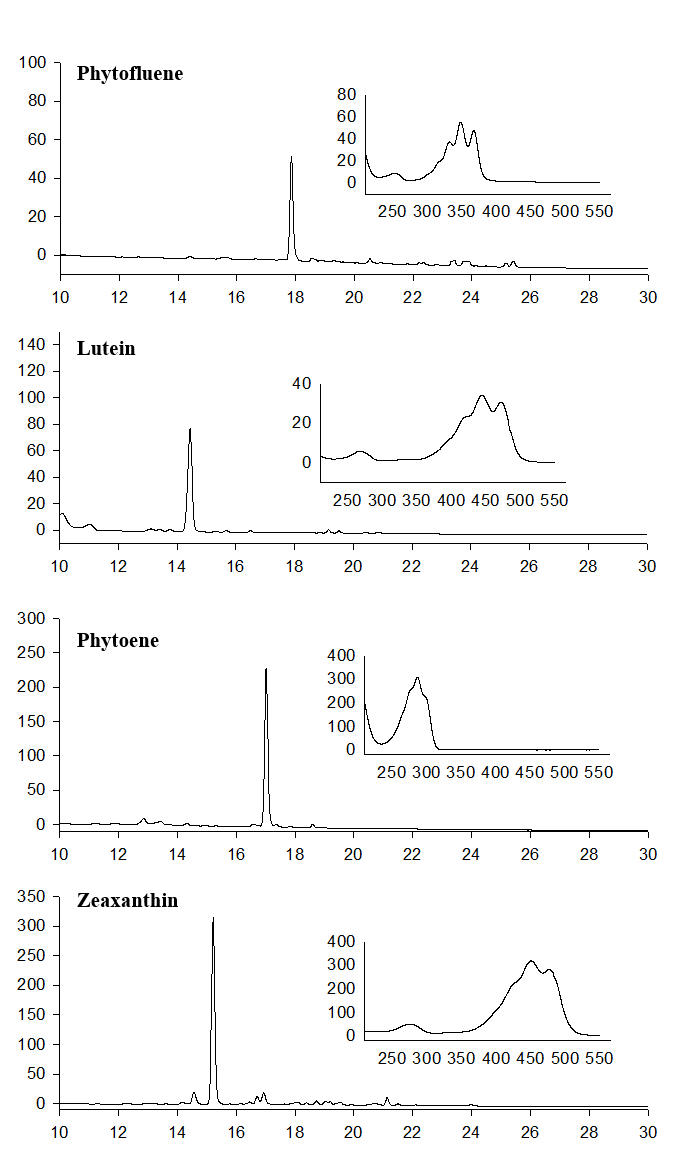


Figure S1: HPLC chromatogram of analytical standards (chlorophyll, β-carotene, lycopene, capsanthin, phytofluene, and lutein) and carotenoid producing *E. coli* extracts (phytoene, zeaxanthin).


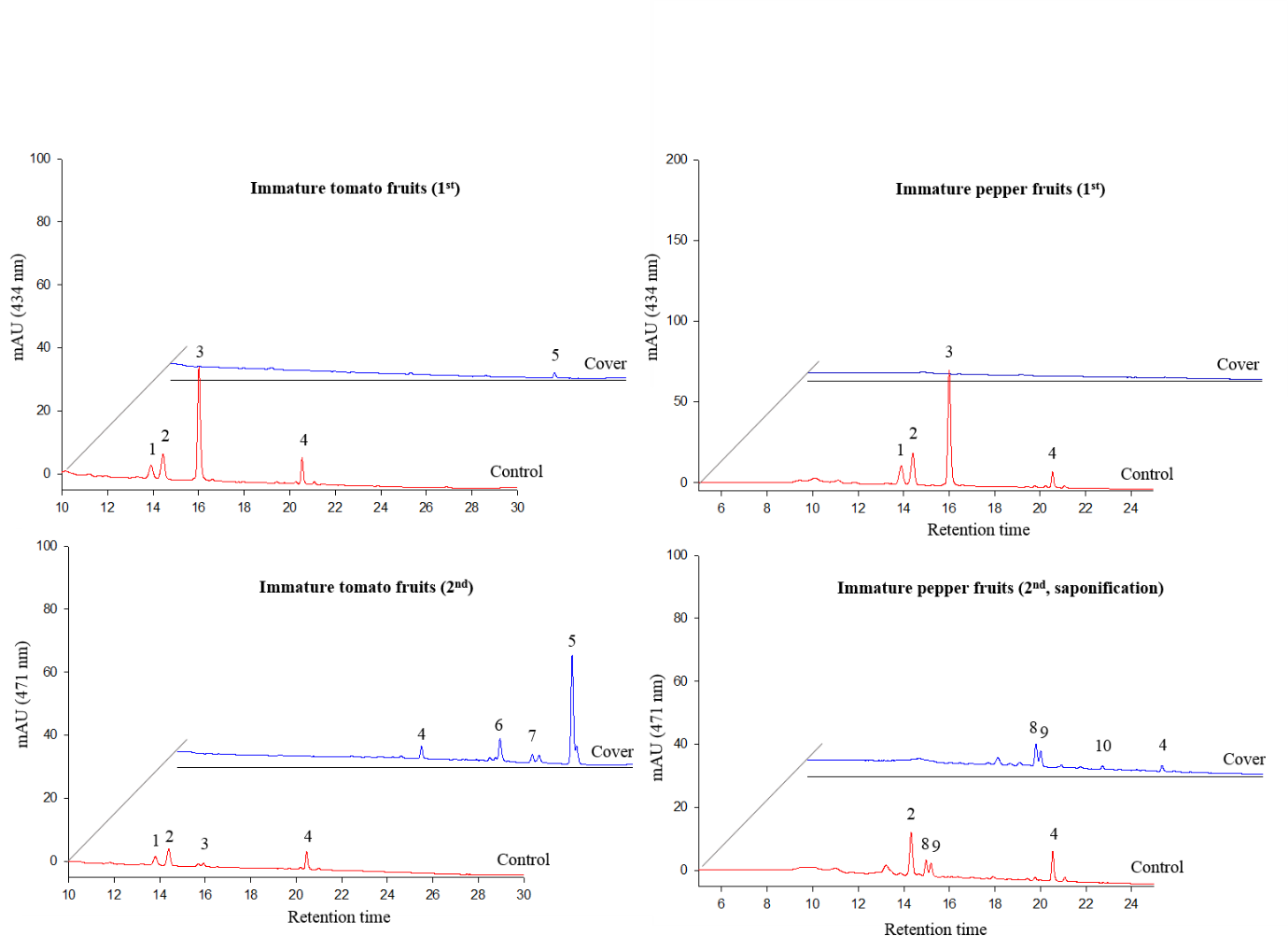


Figure S2: HPLC chromatogram of chlorophylls and carotenoids detected at 434 and 471 nm in immature tomato and pepper fruits. Each number indicates that 1, chlorophyll b; 2, lutein; 3, chlorophyll a; 4, β-carotene; 5, trans-lycopene; 6, γ-carotene; 7, cis-lycopene; 8, capsanthin; 9, zeaxanthin; 10, β-cryptoxanthin.


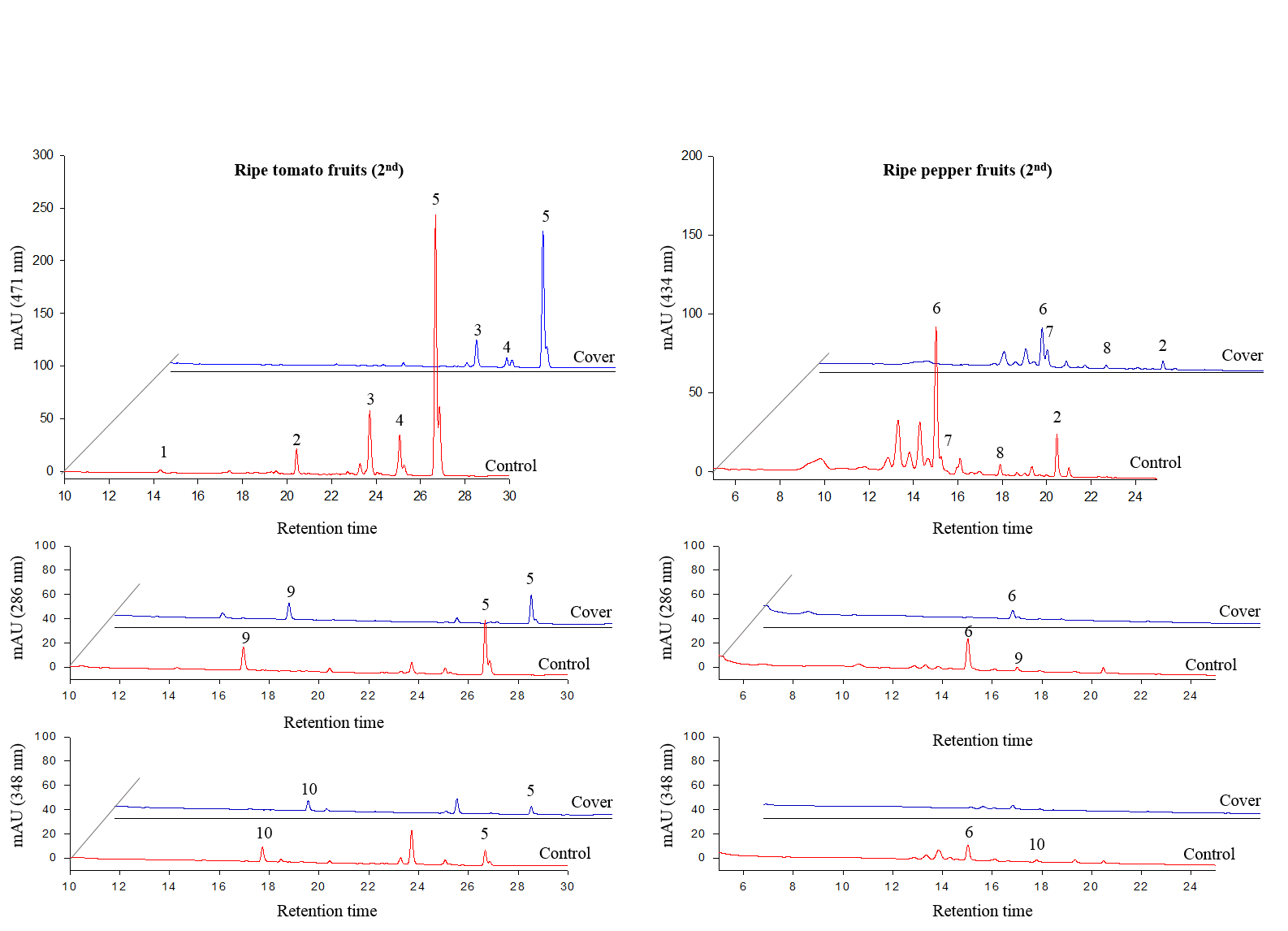


Figure S3: HPLC chromatogram of carotenoids detected at 471, 286, and 348 nm in ripe tomato and pepper fruits. Each number indicates that 1, lutein; 2, β-carotene; 3, γ-carotene; 4, cis-lycopene; 5, trans-lycopene; 6, capsanthin; 7, zeaxanthin; 8, β-cryptoxanthin; 9, phytoene; 10, phytofluene.


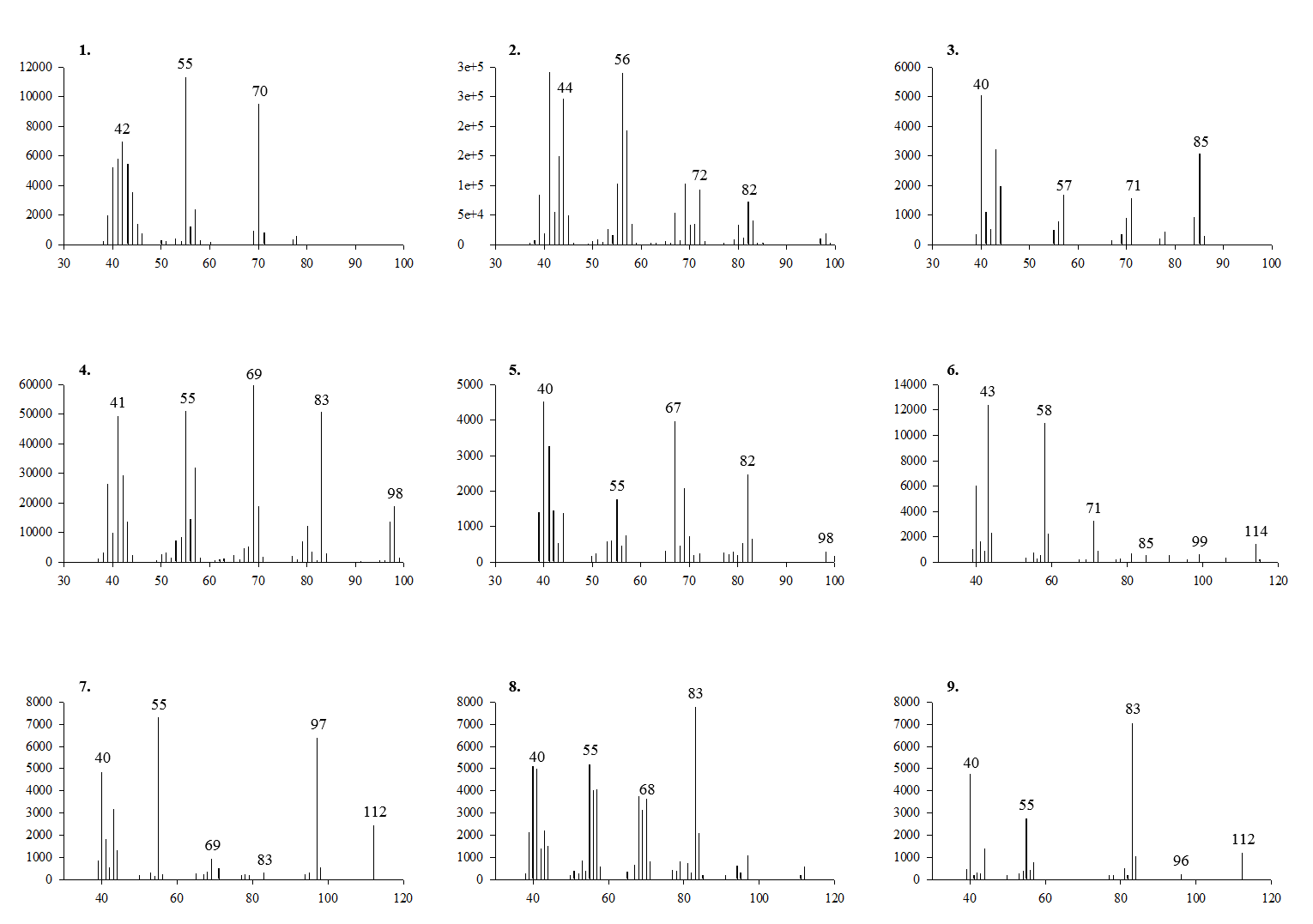


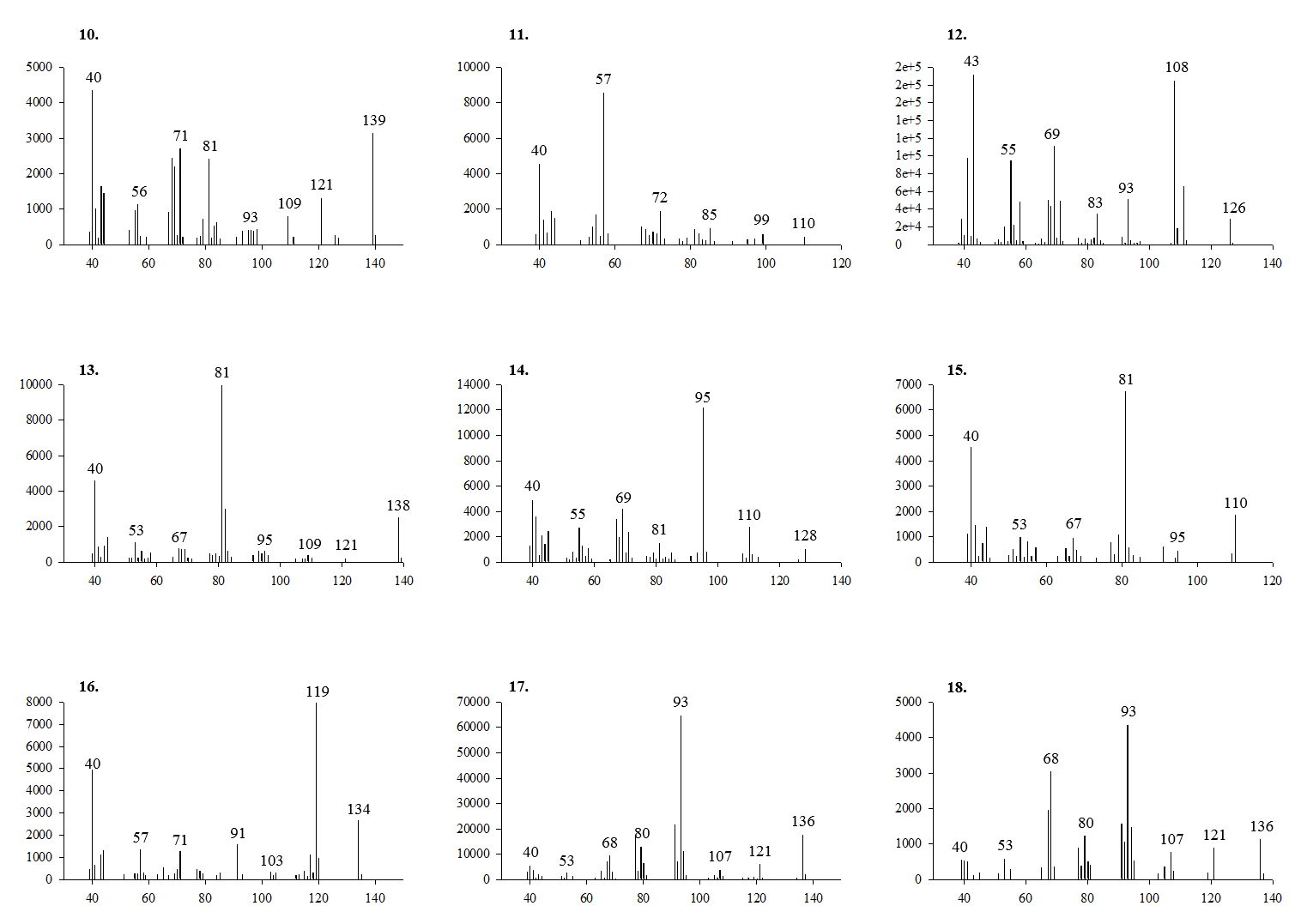


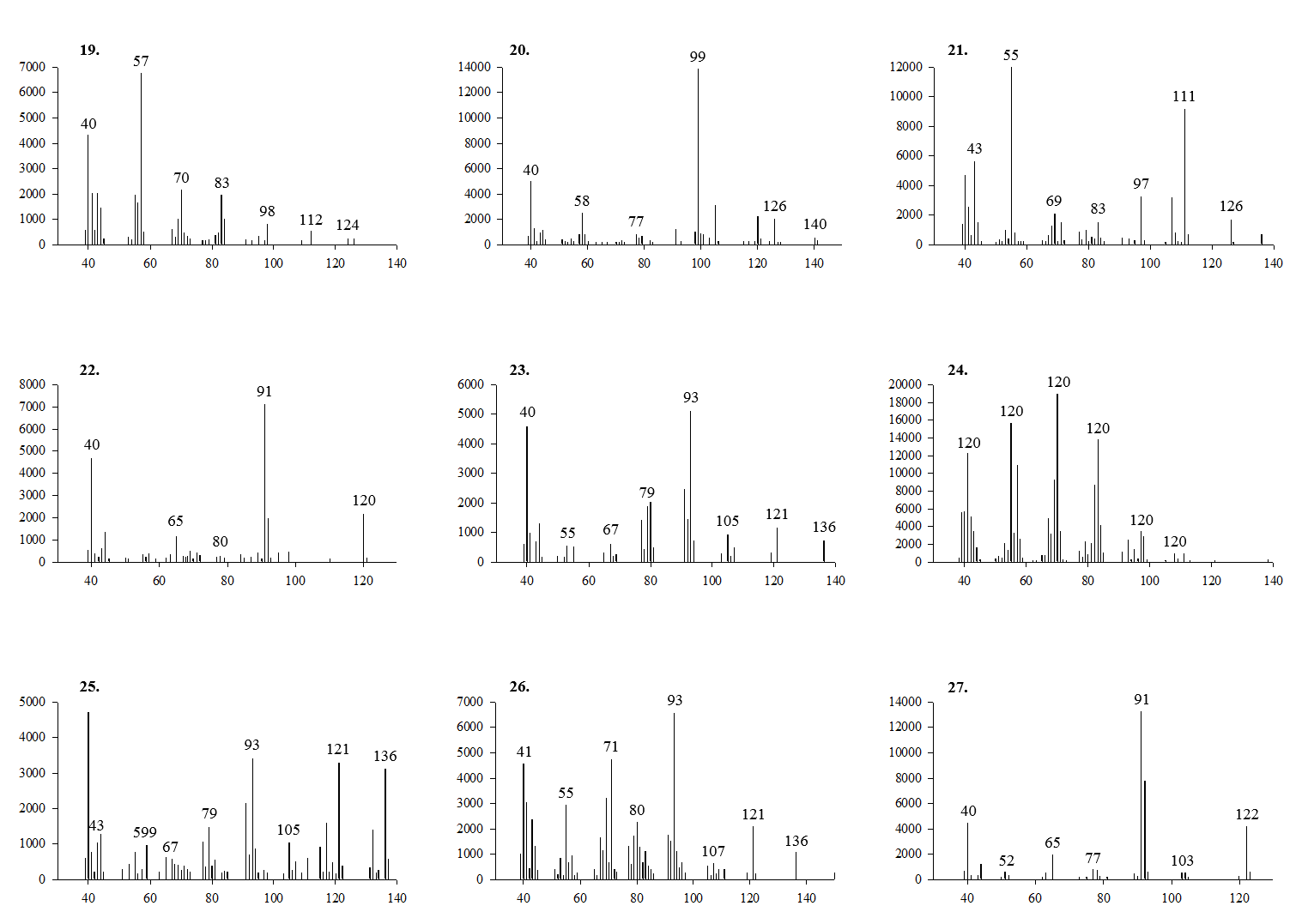


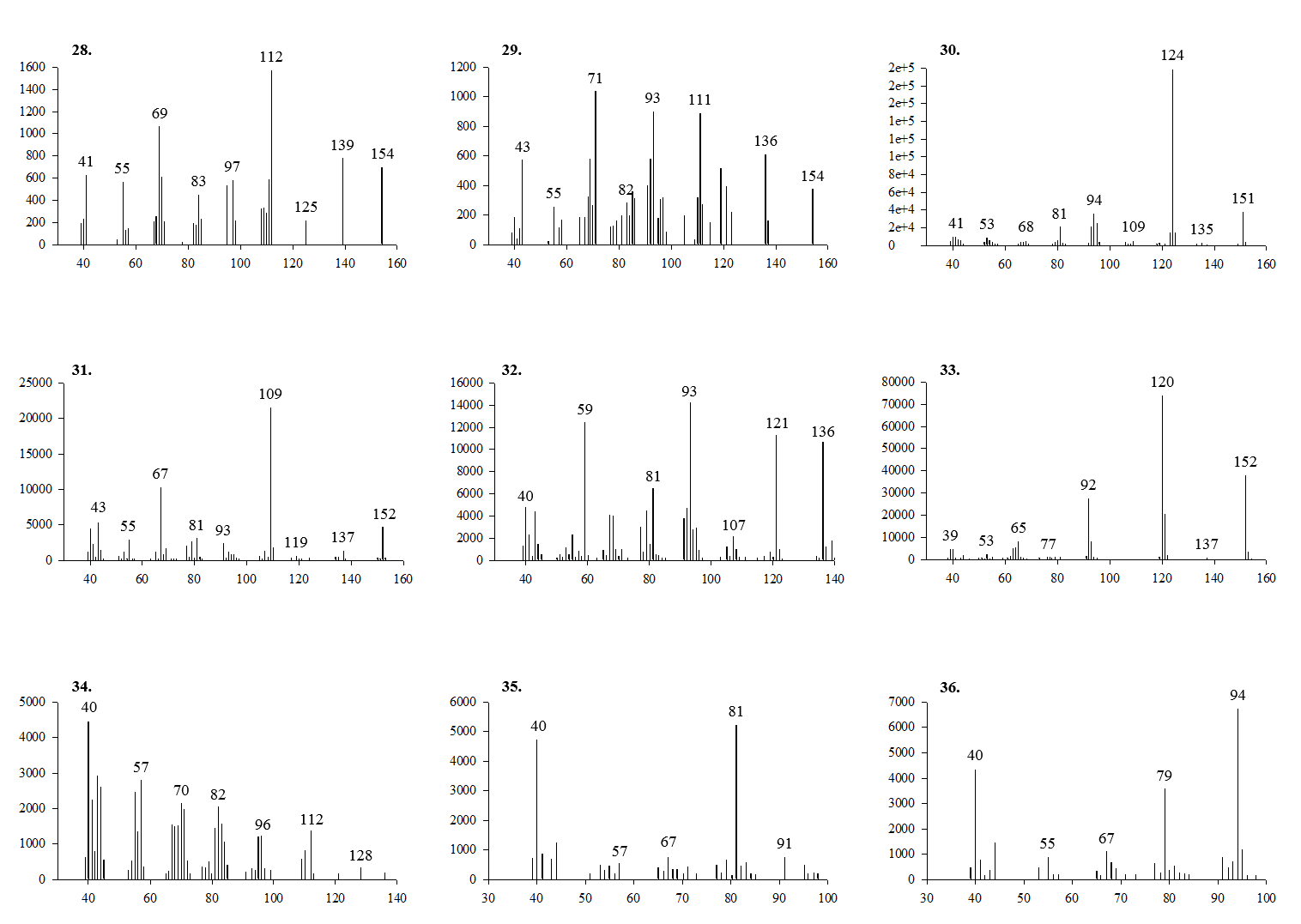


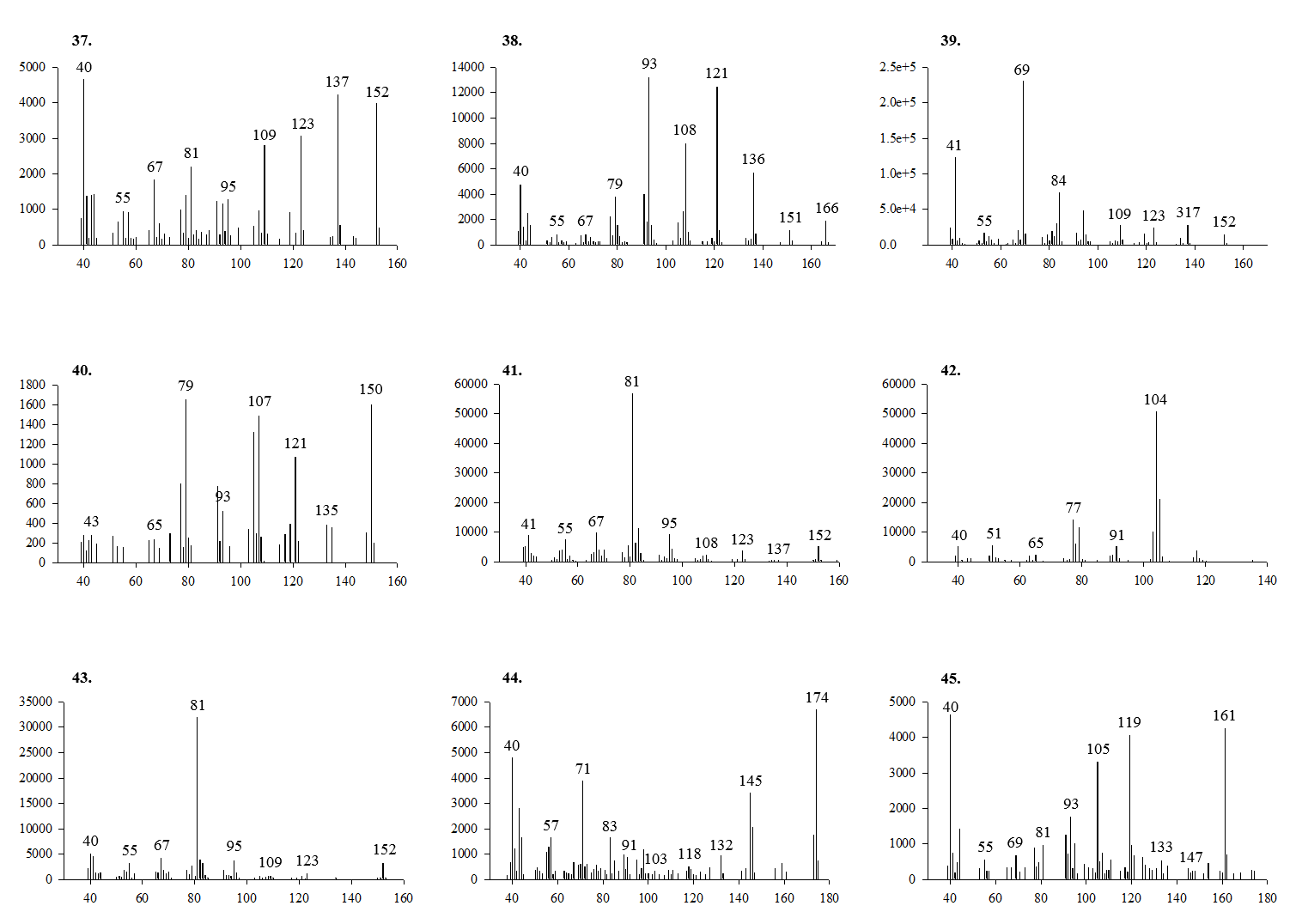


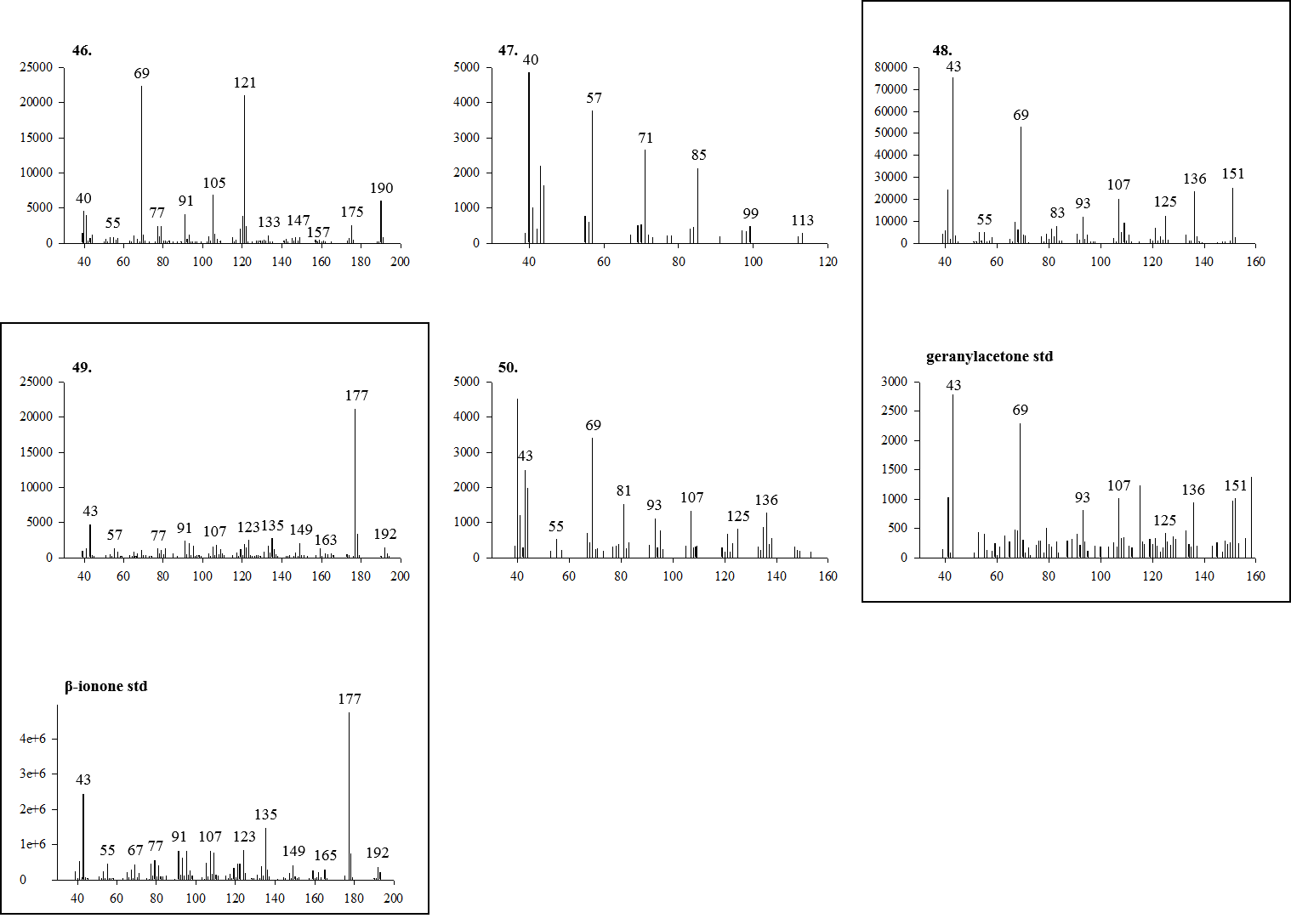


Figure S4: Mass spectrum of volatile compounds listed in Table S2 and analytical standards of β-ionone and geranlyacetone.
